# Long‐term surveillance reveals hybridization by nuclear reassortment and intercontinental spread as major evolutionary drivers in wheat yellow rust

**DOI:** 10.1111/nph.71300

**Published:** 2026-06-14

**Authors:** Mogens Støvring Hovmøller, Tine Thach, Julian Rodriguez‐Algaba, Jens Grønbech Hansen, Marcel Meyer, David P. Hodson, Kumarse Nazari, Robert F. Park, Rita Tam, Mareike Möller, Benjamin Schwessinger, John P. Rathjen, Paula Silva, Venancio Riella, Annemarie Fejer Justesen

**Affiliations:** ^1^ Department of Agroecology, Global Rust Reference Center Aarhus University Slagelse 4200 Denmark; ^2^ Crop Science, Institute of Crop Science and Resource Conservation University of Bonn Bonn 53115 Germany; ^3^ CIMMYT Nepal Khumaltar Lalitpur 44700 Nepal; ^4^ Turkey‐ICARDA Regional Cereal Rust Research Center Izmir 35661 Turkey; ^5^ Plant Breeding Institute, School of Life and Environmental Science The University of Sydney Cobbitty NSW 2570 Australia; ^6^ Research School of Biology The Australian National University Canberra ACT 2601 Australia; ^7^ Instituto Nacional de Investigación Agropecuaria (INIA) Colonia 70000 Uruguay

**Keywords:** global surveillance, haplo‐phased genome, hybridization, *Puccinia striiformis*, SSR haplotype, trajectory simulations, world‐wide spread

## Abstract

- Evolutionary forces affecting crop pathogens, including hybridization and long‐distance dispersal (LDD), may have strong implications for food security and sustainable plant disease control at global scales. However, consolidated evidence is often lacking due to the absence of consistent pathogen surveys beyond national capacities.
- Our study documents world‐wide connectivity between populations of *Puccinia striiformis*, causing yellow rust on cereals and grasses, when analyzing 3240 pathogen samples collected in 41 countries on six continents from 2009 to 2023. Our analyses revealed 10 cases of intercontinental spread of *Puccinia striiformis*, including seven cases with major impact on disease epidemics in recipient areas.
- Somatic hybridization by nuclear reassortment between co‐existing multilocus genotypes (MLGs) on a common host was the most plausible mechanism for the emergence of three novel clonal groups that were first detected in Europe. Subsequently, onward spread to South America and Australia was observed. Several high‐impact incursions from South Asia into East Africa were also detected, including a genotype with a dramatic impact on wheat breeding programs of global relevance.
- Our study stresses an urgent need for coordinated crop pathogen monitoring across borders. Only global efforts will enable prevention and control of airborne pathogens that represent major challenges for food security at regional and global scales.

## Graphical abstract

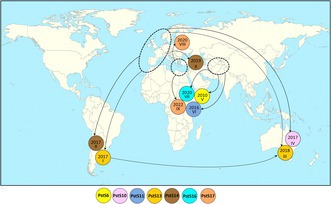

## Introduction

An increase in human travel and trade and the escalating speed of changing climate and weather patterns have renewed attention on disease epidemiology in humans, animals and crop plants, for example by the US National Academies of Sciences (Olsen *et al*., 2011), and the United Nations Food and Agriculture Organisation (FAO, 2024). Long‐distance dispersal (LDD) is an important epidemiological feature of many pathogens, including those affecting crops, because even rare LDD events can have a major impact on crop production (Brown & Hovmøller, 2002; Carvajal‐Yepes *et al*., 2019). Recent reviews suggest that LDD events of cereal rust pathogens are increasing in frequency at the intercontinental scale, possibly due to intensified crop production, climate change resulting in milder winters in temperate regions, and increased travel and commerce (Park, 2015; Jin *et al*., 2020; Hovmøller *et al*., 2023). More frequent LDD events may delay or prevent achievement of the 70% increase in food and agricultural production required by 2050 to meet global population growth (van Dijk *et al*., 2021).

One major obstacle for the detection of LDD events in plant pathology is the lack of regular and extensive pathogen population surveys beyond national capacities (Jeger & Pautasso, 2008; Ristaino *et al*., 2021), including common sampling protocols, harmonized population genetic data and analytic tools (Patpour *et al*., 2022). This makes it difficult to establish the exact geographical and evolutionary origins of a new pathogen variant as it spreads into recipient areas. Multiple tools are available for analyses of population genetic features, such as genetic distance, population subdivision and migration rates within and among pathogen populations (Zhan *et al*., 2003; Bebber *et al*., 2014; Ali *et al*., 2014a). In predominantly clonal populations with a high degree of preserved haplotypes, the tracking of individual multilocus genotypes (MLGs) may represent a different and powerful strategy for the detection of LDD events (Brown & Hovmøller, 2002; Hovmøller *et al*., 2002).

The rust fungi (*Pucciniales*) are the most species rich and complex order of plant pathogens, consisting of almost 8000 species. They are characterized by a true biotrophic lifestyle and are individually adapted to their host plant at species and cultivar levels (Flor, 1971; Anikster, 1985; Aime *et al*., 2017). *Puccinia striiformis* f.sp. *tritici* (*Pst*), causing yellow (or stripe) rust on wheat, is a dikaryotic, heteroecious and macrocyclic fungus requiring a primary cereal/grass host and a taxonomically unrelated alternate host, for example *Berberis* spp. to complete its life cycle (Jin *et al*., 2010).

Multiple studies have reported high diversity in *Pst* populations in the near‐Himalayan region (including China, Pakistan and Nepal), which is considered the center of diversity of this fungus (Duan *et al*., 2010; Ali *et al*., 2014b; Thach *et al*., 2016; Huang *et al*., 2021); however, the functional role of the sexual host, *Berberis* spp., in these areas is not fully understood because *Pst* is rarely observed on *Berberis* spp. in nature (Jin *et al*., 2010; Berlin *et al*., 2013; Wang *et al*., 2016; Rodriguez‐Algaba *et al*., 2022).

By contrast, many studies in other regions have documented a clonal *Pst* population structure, where spontaneous and high mutation rates coupled with host‐induced selection are major evolutionary driving forces, for example in Europe (Hovmøller *et al*., 2002, 2016; Enjalbert *et al*., 2005; Hovmøller & Justesen, 2007; de Vallavieille‐Pope *et al*., 2012; Saunders *et al*., 2019), Australia (Wellings & McIntosh, 1990; Steele *et al*., 2001; Ding *et al*., 2021), South America (Anibal Carmona *et al*., 2019), North America (Markell & Milus, 2008; Brar *et al*., 2018), as well as Central‐ and West Asia and North‐ and East Africa (Hovmøller *et al*., 2008; Ali *et al*., 2014a, 2017; Thach *et al*., 2016; Walter *et al*., 2016). Several studies involved comparative analyses between high‐diversity areas in the near‐Himalayan region, including China and clonal populations from elsewhere, revealing clear geographic population structures with limited overlap. At the same time, rare LDD events have ensured some degree of connectivity across large time and spatial scales (Hovmøller *et al*., 2008; Ali *et al*., 2014a; Anibal Carmona *et al*., 2019; Ding *et al*., 2021; Župunski *et al*., 2024). In 2011, clonal populations in Europe were influenced by incursions likely originating from populations in the near‐Himalayan region, including a number of distinct MLGs with very different virulence‐ and molecular patterns compared with the pre‐existing population that were largely replaced within a few years (Hovmøller *et al*., 2016).

So far, somatic hybridization by fusion of hyphae, which may lead to nuclear reassortment where parental haplotypes are preserved, has only been reported under experimental conditions in *Pst* (Little & Manners, 1967, 1969; Lei *et al*., 2017), unlike for the wheat leaf rust pathogen *P. triticina* (Park & Wellings, 2012; Sperschneider *et al*., 2023), the wheat stem rust pathogen *P. graminis* f.sp. *tritici* (Li *et al*., 2019), and *P. coronata* causing oat crown rust (Henningsen *et al*., 2024).

The increased focus on *Pst* epidemiology at the global level since 2000 coincided with the emergence of *P. graminis* f.sp. *tritici* (*Pgt*) race ‘Ug99’ in East Africa that was virulent on more than 80% of wheat lines developed by The International Maize and Wheat Improvement Center (CIMMYT; McIntosh & Pretorius, 2011). In response, the Borlaug Global Rust Initiative (BGRI) was formed and laid the foundation for more extensive and regular wheat rust disease and pathogen surveys. These included Africa and Asia, which were connected with national rust survey programs in Europe and elsewhere by the establishment of the Global Rust Reference Center (GRRC) in 2008 (Hovmøller, 2011). This resulted in commonly agreed sampling and genotyping methodologies for wheat yellow‐ and stem rust, often using a core set of simple sequence repeat (SSR) or single‐nucleotide polymorphic (SNP) markers (Bahri *et al*., 2008; Patpour *et al*., 2022; Szabo *et al*., 2022; Thach *et al*., 2025), and more recently MARPLE (Radhakrishnan *et al*., 2019; Župunski *et al*., 2024), which have allowed a high degree of alignment of results from studies representing different time periods and sampling areas.

The combined international efforts have allowed analyses of the spread and evolution of *Pst* and *Pgt* across borders and continents, including the return of *Pgt* into Europe and connectivity to epidemics elsewhere (Patpour *et al*., 2022), including Western Siberia 2015–2016 (Shamanin *et al*., 2016) and East Africa (Olivera *et al*., 2015). In addition, independent surveys have reported large‐scale epidemiological consequences of new incursions of *Pst* into South America in 2017 (Anibal Carmona *et al*., 2019; Riella *et al*., 2024) and Australia in 2017–2018 (Ding *et al*., 2021).

The present study investigated drivers of *P. striiformis* evolution at a global scale based on genotypic analyses of 3240 samples; 3180 samples collected across four continents (37 countries) between 2009 and 2023, complemented by genotype information of an additional 60 samples representing long‐term surveillance efforts in Europe, North America (Milus *et al*., 2015a) and Australia (Ding *et al*., 2021). LDD events were identified by investigating the potential sharing of genotypes in putative source and recipient areas, considering the time of first detection as well as the accumulation of genetic diversity in the considered populations. In addition, the plausibility of wind dispersal along hypothesized transmission routes was explored by conducting trajectory simulations considering typical spore survival times and mean wind patterns. The likely evolutionary origin of successful LDD migrants was explored in detail in specific cases, including hypothetical hybridization events by nuclear reassortment. The prospects for prevention and mitigation efforts to reduce the increasing impact of crop pathogen spread at an intercontinental scale are discussed.

## Materials and Methods

### Collection and recovery of yellow rust samples

Pathogen sampling reflected the coordinated efforts in wheat rust surveillance facilitated by research programs within the Borlaug Global Rust Initiative and European Union research and network initiatives, collectively defining seven geographic populations of *Puccinia striiformis* f.sp. *tritici* Erikss (*Pst*) between 2009 and 2023 as well as reference populations in North America and Australia, the latter representing the main clonal divergences since first incursion into Australia in 1978 (Supporting Information Fig. S1).

A total of 3180 time‐ and geo‐referenced samples of yellow rust mainly from wheat (and occasionally other cereals and grasses) were collected in 37 countries on four continents between 2009 and 2023 (Table S1). The number of samples varied across years and locations, reflecting the prevalence of epidemic outbreaks and available sampling efforts. The Regional Cereal Rust Research Center (RCRRC), ICARDA, Turkey, provided 213 samples from the Middle East and West Asia. An additional pre‐existing 60 isolates served as references of previously identified *Pst* groups and *Pst* populations in the United States (Milus *et al*., 2015b) and Australia (Ding *et al*., 2021), in total resulting in 3240 samples.

Recovery of incoming samples and multiplication of urediniospores followed the procedure by Thach *et al*. (2025), after which they were harvested, dried and stored in liquid nitrogen (−196°C) or freezer (−80°C) until further use.

### DNA extraction and SSR genotyping

From 2009 to 2012, DNA was extracted from dried urediniospores (*c*. 20 mg per sample) according to Thach *et al*. (2016). Since 2013, extraction was based on dried leaf segments with single rust lesions taken from multiplication plants or directly from incoming samples according to Thach *et al*. (2025). Genotyping was done using 19 SSR co‐dominant markers and allele sizes were manually scored, allowing up to two missing markers per sample (Thach *et al*., 2025). Samples with allele sizes and/or combinations suggesting more than a single genotype were excluded from the dataset. Detection of novel or exotic genotypes in a region/country was confirmed by additional independent DNA extractions from original sample material and genotyping. Two hundred and thirteen samples from the Middle East (2018–2020) collected by Turkey‐ICARDA RCRRC were SSR genotyped by EUROFINS, France. Scoring of allele sizes and data alignment to existing format were carried out at the GRRC. Assignment of MLG (MLG #) was done for samples without missing data using poppr package v.2.9.3 implemented in the R environment (Kamvar *et al*., 2014, 2015); for samples with missing data at a maximum of two marker loci, new MLGs were considered in case scored allele sizes revealed additional variability, whereas missing observations were not taken into account for MLG identity. Duplicate MLGs derived from the same incoming sample were removed from the dataset.

### Clustering within clonal groups

Clustering within 19 previously defined clonal *Pst* groups (PstS0–PstS18) comprising closely related MLGs and associated races (Thach *et al*., 2025) was used to assess population differentiations across geographical regions and over time. The definition and naming of new clonal groups were guided by evidence of significant divergence from previously described groups, the presence of group‐specific diagnostic markers and/or documented epidemic impact across large geographic areas (Walter *et al*., 2016; Ali *et al*., 2017). An updated summary of named *Pst* groups is available at the GRRC website: https://agro.au.dk/forskning/internationale‐platforme/wheatrust/yellow‐rust‐tools‐maps‐and‐charts/definitions‐of‐races‐and‐genetic‐groups.

### Population diversity and phylogenetic analysis

The discriminative power of the SSR marker set was evaluated using a genotype accumulation curve implemented in the R package poppr (v.2.9.3) based on samples without missing data (*n* = 2588), and 10 000 random resampling iterations without replacement (Kamvar *et al*., 2014; Fig. S2).

For phylogenetic analysis, the dataset was clone‐corrected by including one representative isolate per MLG. An unrooted phylogenetic neighbor joining tree was constructed to visualize global genetic diversity and the positions of major clonal groups (PstS0–PstS18). Genetic distances were calculated using Bruvo's distance for SSR data under a stepwise mutation model (Bruvo *et al*., 2004) implemented in poppr R package v.2.9.3. Node support was assessed using 2130 bootstrap replicates and only values ≥ 75 were retained (Kamvar *et al*., 2014).

### Tests for somatic hybridization by nuclear reassortment

The hypothesis of somatic hybridization via nuclear reassortment among putative parental isolates was explored through a series of analytical steps (Fig. S3), taking advantage of haplotype‐phased chromosome‐scale genome assemblies. These included as follows: (1) a PstS0 isolate (Plant Breeding Institute accession no. 415 collected in 1982; Schwessinger *et al*., 2018; Tam *et al*., 2025), (2) Australian PstS10 isolate (Ding *et al*., 2021) generated as described previously for PstS0 (Tam *et al*., 2025) with the exception of using PacBio HiFi and Oxford Nanopore Technology (ONT) R10.4 based long reads and Hi‐C as input for Verkko genome assembler (Rautiainen *et al*., 2023), (3) a reference genome of PstS7 used ONT R10.4.1 simplex reads and Hi‐C with hifiasm ‐‐ont (Cheng *et al*., 2025) and (4) a chromosome‐scale assembly of an isolate of the PstS1_2 Lineage (Schwessinger *et al*., 2022). Assembly quality statistics are provided in Table S2.

Nuclear‐specific chromosome‐anchored SSR haplotypes of hypothetical parental and hybrid isolates were determined by blast analysis (‐word_size 12 ‐evalue 1 ‐dust no ‐outfmt 6) of 20 primer sequence pairs against the haplotype‐phased genomes of PstS0, PstS7, PstS10 and PstS1_2 (Table S3) and visualized according to Wolfe *et al*. (2013). The calculated SSR haplotypes were aligned with observed (scored) allele sizes (Table S3) to allow independent validation of hypothesized parental and hybrid haplotypes of PstS10, PstS13, PstS14 and PstS18, respectively.

To further validate the proposed hybrid PstS10 and two hypothetical donors, PstS0 and PstS7, pairwise whole‐genome alignments were conducted using nucmer v.3.1 as part of the mummer package (Marçais *et al*., 2018) with default settings. Only alignment blocks with > 75% identity and longer than 500 bp were retained. SNP counts and average identity were computed from 1‐to‐1 alignments using mummer's DNAdiff v.1.3. Dot plots were generated using a script adapted from paf2dotplot.r (https://github.com/moold/paf2dotplot).

### Virulence phenotyping

Recovered, hypothesized migrant isolates from putative source and recipient areas were virulence phenotyped to provide an independent assay for genetic relatedness. An updated protocol using a 0–9 disease scoring scale according to McNeal *et al*. (1971), where infection types (IT) from 0 to 6 were generally considered incompatible (avirulent) and IT from 7 to 9 compatible (virulent) was followed (Thach *et al*., 2025). The interpretation of virulence/avirulence was generally based on two (or more) independent wheat lines carrying the considered *P. striiformis Yr* resistance gene.

### Plausibility of wind dispersal

Plausibility of wind dispersal was explored for seven hypothesized dispersal routes reflecting observational data in the study, that is (1) Europe – South America, (2) South America – Australia, (3) Europe – Australia, (4) Central/South Asia – East Africa, (5) Middle East – Europe, (6) Middle East – East Africa and (7) Europe/NW Africa – Middle East, respectively. The analyses were based on (1) reviewing data from previous studies on related fungal pathogens and other microorganisms in similar geographies (Brown & Hovmøller, 2002; Isard *et al*., 2005, 2011; Meyer *et al*., 2017a,b; Prank *et al*., 2019; Bradshaw *et al*., 2022; Radici *et al*., 2022; Brodsky *et al*., 2023; Miedaner & Garbelotto, 2024); (2) long‐term mean wind direction and speed at times of overlapping wheat seasons at source and recipient areas (https://iridl.ldeo.columbia.edu/maproom/Global/Climatologies/Vector_Winds.html); (3) sensitivity analyses for 5 and 10 d spore survival time during atmospheric transport from the literature (Maddison & Manners, 1972; Rapilly, 1979; Aylor, 1986); and (4) conducting a set of atmospheric trajectory simulations using HYSPLIT (Stein *et al*., 2015) with two different global‐scale gridded meteorological datasets (NOAA's GDAS, 3‐hourly temporal resolution and 1 degree horizontal spatial resolution; ECMWF's ERA5, hourly temporal resolution and 0.25 degree spatial resolution (Hersbach *et al*., 2020)). Time‐backwards trajectory simulations were conducted from locations in recipient areas with GDAS meteorological input data to investigate origins of air masses that could have transported spores to recipient areas where they were first detected. Time‐forward simulations were conducted with ERA5 meteorological input to analyze the plausibility of stepwise airborne transmission along two selected pathways (Europe to South America via Northern Africa; and Central Asia to East Africa via the Middle East).

## Results

### Genotypic *Pst* diversity at global scales

**Fig. 1:**
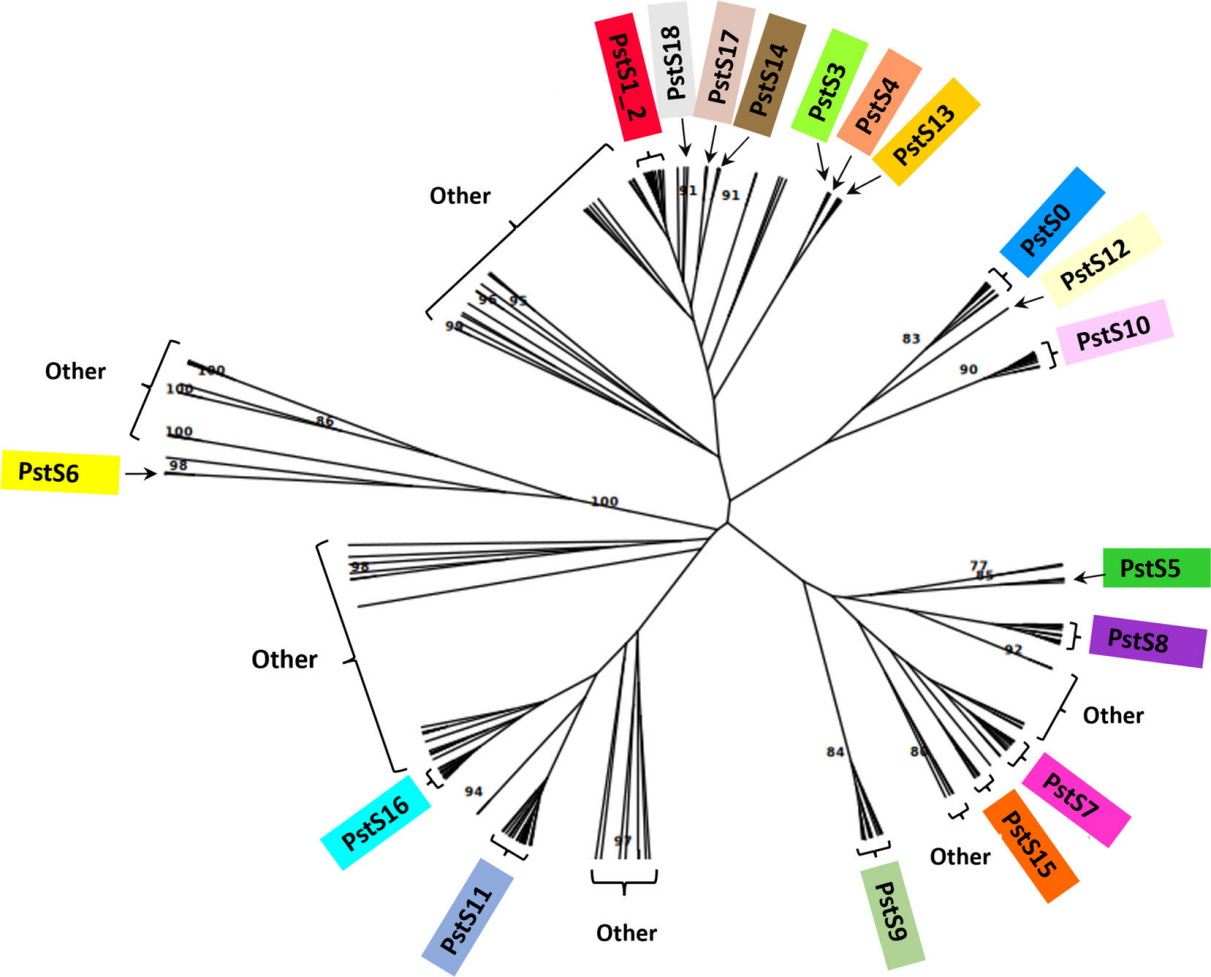
Unrooted neighbor joining tree (clone‐corrected) using Bruvo's distance showing the genetic relationship of 244 multilocus genotypes (MLGs) representing world‐wide sampling of *Puccinia striiformis* in 41 countries on six continents (2009–2023), including reference isolates representing populations in Australia (1978–2021) and North America (1972–2023). Position of 18 named clonal groups (PstS0–PstS18) is indicated, each group previously defined based on divergence from pre‐existing groups/MLGs and concurrently named in case of significant impact on disease epidemiology over larger areas in multiple years. Group colors are aligned with Fig. 2, where prevalence over time and area of individual named groups are shown. These typically consisted of clusters of closely related MLGs, whereas individual branches generally represented MLGs from diverse populations in South Asia and Central/West Asia. Values based on 2130 bootstraps with a value of 75% are shown.

A majority of the samples investigated (91.7%) grouped into previously defined clonal groups PstS0–PstS18 (Fig. 1). The remaining samples consisted of 74 diverse MLGs mainly from South Asia, where high diversity has been reported previously, and from non‐wheat hosts in northern Europe, for example barley, wild grasses and triticale. The 20 reference samples from North America comprised six MLGs distributed within PstS0, PstS1_2 and PstS18, and two unnamed groups. The 31 reference samples from Australia were embedded in seven MLGs that grouped within PstS0, PstS1_2, PstS10 and PstS13, respectively, whereas six reference samples from Europe and one from East Africa (1960–2006) represented PstS1_2, PstS3 and PstS4.

Sample numbers of individual clonal groups (PstS0–PstS18) varied considerably, reflecting their overall prevalence (Table S4). Also, the number of MLGs within each group varied significantly, likely reflecting time since first detection as well as disease prevalence and related sampling intensity. The highest number of MLGs was observed for PstS1_2, first detected in East Africa in the 1970s, and PstS11 that was first detected in South Asia in 2012. Often, the most frequent MLG accounted for 40% or more of the samples within a group. In relation to hybridization and LDD, it was also the predominant MLG that was involved (Tables 1, S4).

**Table 1:** Intercontinental spread of multilocus genotypes (MLGs) of *Puccinia striiformis* clonal groups – first detection in source area and recipient areas.

Dispersal event cf. (Fig. 5)	Migrant (MLG no.)	SSR clonal group	First detected (population and year)	References	Recipient population and year	Race confirmation in source and recipient population^3^
I	21	PstS13	Europe (2015)	Ali *et al*. (2017)	South America (2017)^2^	‐,2,‐,‐,‐,6,7,8,9,‐,‐,‐,‐,‐,‐,‐,‐,AvS,‐
II	134	PstS14	Europe/NW Africa (2015–2016)	Hovmøller (2011)^1^	South America (2017)^2^	‐
III	21	PstS13	Europe (2015)	Ali *et al*. (2017)	Australia (2018)^2^	‐
IV	244	PstS10	Europe (2012)	Ali *et al*. (2017)	Australia (2017)^2^	‐
V	8	PstS6	Central Asia (2009)	Ali *et al*. (2017)	East Africa (2010)^2^	1,2,‐,‐,‐,6,7,‐,9,‐,‐,17,‐,‐,27,‐,‐,AvS,‐
VI	78	PstS11	Central Asia (2012)	Hovmøller *et al*. (2008)^1^	East Africa (2016)^2^	‐,2,‐,(4),‐,6,7,8,‐,‐,‐,17,‐,‐,27,32,‐,AvS,‐
VII	205	PstS16	South Asia (2017)	Hovmøller *et al*. (2023)^1^	East Africa (2020)^2^	1,2,3,(4),‐,6,7,8,9,‐,‐,17,‐,25,27,32,‐,AvS,‐
VIII	170	PstS17	Middle East (2018)	Hovmøller *et al*. (2016)^1^	Europe (2020)	‐,2,‐,‐,‐,6,7,8,‐,‐,‐,17,‐,‐,‐,32,(Sp),AvS,Amb
IX	170	PstS17	Middle East (2018)	This paper	East Africa (2022)	‐,2,‐,‐,‐,6,7,8,‐,‐,‐,17,‐,‐,‐,32,(Sp),AvS,Amb
X	138	PstS14	Northwest Africa (2016)/Europe (2016)	This paper	Middle East (2019)	‐

^1^ Annual reports from Global Rust Reference Center (accessible: www.wheatrust.org).

^2^ High‐impact event in recipient areas.

^3^ Figures and symbols designate virulence and avirulence (‐) corresponding to *Pst* resistance genes: *Yr1, Yr2, Yr3, Yr4, Yr5, Yr6, Yr7, Yr8, Yr9, Yr10, Yr15, Yr17, Yr24, Yr25, Yr27, Yr32* and the resistance specificity of Spalding Prolific (Sp), Avocet S (AvS) and Ambition (Amb).

**Fig. 2:**
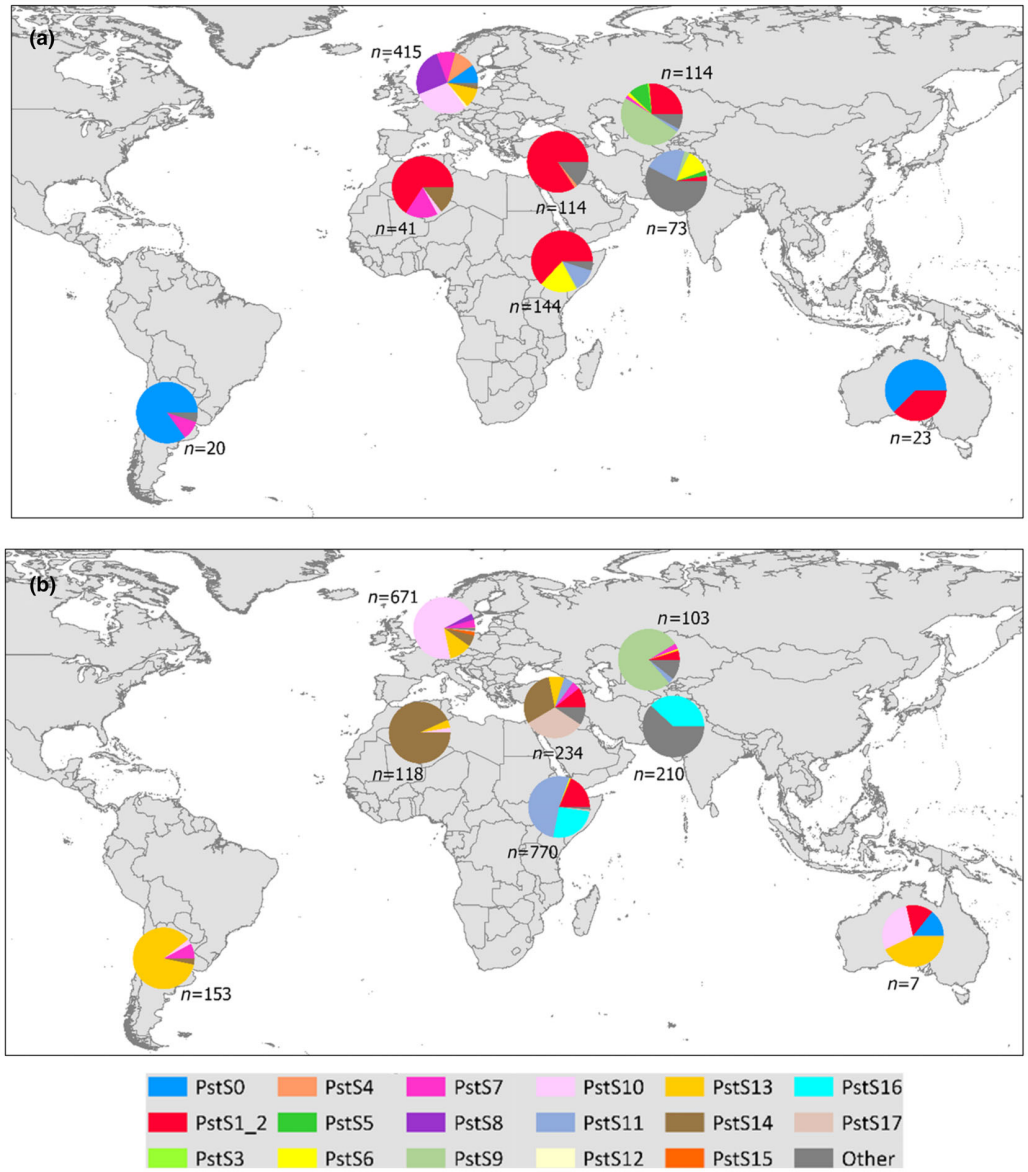
Frequency of *Puccinia striiformis* clonal groups in distinct geographical populations covering major wheat growing regions world‐wide between 2009 and 2023. (a) Sampling period 2009–2016, and (b) sampling period 2017–2023, (*n* = sample size). Australian data represent key reference samples 1978–2021, that is from first *Pst* disease appearance on the continent and until recent time. North American reference data not shown due to low sample size and uneven representation over time and area.

Significant changes in prevalence over time and across geographical areas (Northern Europe, Middle East, East Africa, Northwest Africa, South Asia, Central‐West Asia, South America and Australia) were observed, suggesting geographic subdivisions and dynamic pathogen populations over time (Fig. 2).

The diverging geographical distribution of clonal groups was striking; for example, PstS1_2 was prevalent across Africa and the Middle East but absent from South America, Europe and South Asia. PstS5 and PstS9 were only detected in Central Asia, and PstS0 was only detected in Europe, the Americas and Australia. PstS8 was prevalent in northern Europe until 2016 but not detected in other areas. PstS10 was the most prevalent group in Europe from 2014 and onwards but was not detected in East Africa and South‐ and Central Asia.

The dataset revealed several cases of emergence of novel clonal groups with significant epidemiological impact on a global scale. For example, the first detected MLG in PstS10 (MLG #244) became widespread rapidly in Europe, accounting for a total of 82% of PstS10 samples in the study (Table S4). This first detected MLG was followed by the emergence of an additional 14 closely related MLGs, consistent with clonal evolution by mutation. The first MLG in PstS13 (#21) was only detected in Europe during 2015–2016, and was first observed in South America in 2017, where yellow rust re‐appeared at epidemic levels after many years without significant impact. The emergence of a total of six closely related MLGs in PstS13 revealed another case of typical clonal evolution. The first detected MLG of PstS14 in NW Africa (#138, 72% of samples; Table S4) represented a third novel clonal group in the dataset.

### Exploring the hypothesis of somatic hybridization

The first detected MLGs within PstS10, PstS13 and PstS14 diverged significantly from previous clonal groups and MLGs, and they were not resampled outside of Europe/ NW Africa until 2–3 yr after first detection. We therefore explored the hypothesis of evolution by hybridization of co‐existing parental isolates on common host plants and nuclear reassortment according to Fig. S3. An initial screen of isolates of named clonal groups that could potentially contribute to a somatic hybridization resulting in PstS10 MLG #244 (Table S4) revealed that all except PstS0 and PstS7 isolates were excluded due to mismatching allele sizes (Table S5).

**Fig. 3:**
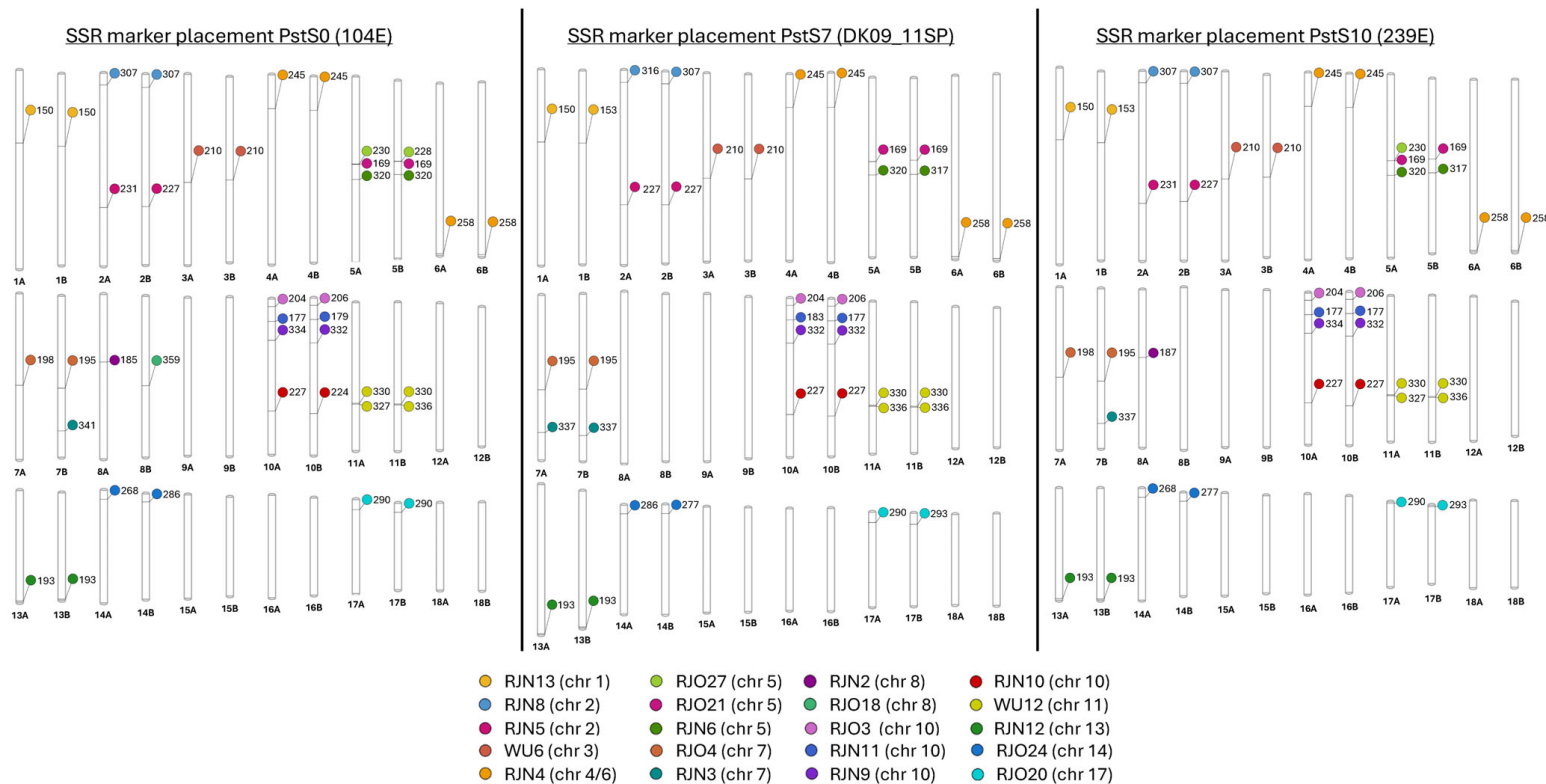
Simple sequence repeat (SSR) marker placement on haplotype‐phased genomes of *Puccinia striiformis* clonal groups PstS0, PstS7 and PstS10 determined by BLAST analyses using pairwise SSR primer sequences. Markers were distributed across 13 chromosomes and detected in both nuclei (denoted a and b) except for RJN2 and RJO27. RJN2 amplifies allele size 185 in Australian PstS0 isolates, whereas allele size 187 is common in PstS0 in Europe, including the hypothesized PstS0 parental isolate. RJO3 (chromosome 10) was generally monomorphic and not scored in this study.

To support our initial observation of PstS0 and PstS7 as potential nuclear donors for PstS10, a comparative analysis of the chromosomal locations of the 19 SSR markers was performed. This analysis made use of existing and newly generated haplotype‐phased reference genome assemblies of PstS0 (Tam *et al*., 2025), PstS7 and PstS10 (this study; Table S3; Fig. 3), respectively.

Interestingly, the SSR markers were distributed on 13 of 18 assigned *Pst* chromosomes, generally detected in both nuclei, except for RJN2, RJN3 and NJO27, resulting in a set of well‐documented markers with a wide representation of the *Pst* genome. The SSR haplotypes derived from genome data, and the corresponding haplotypes deduced from the observed MLGs in the dataset, resulted in a full match based on a historic prevalent MLG #277 of PstS0 in Europe, MLG #321 in PstS7, first detected in Europe in 2011, and the resulting hybrid in PstS10, MLG #244 that was first detected in 2012 (Table S4).

**Fig. 4:**
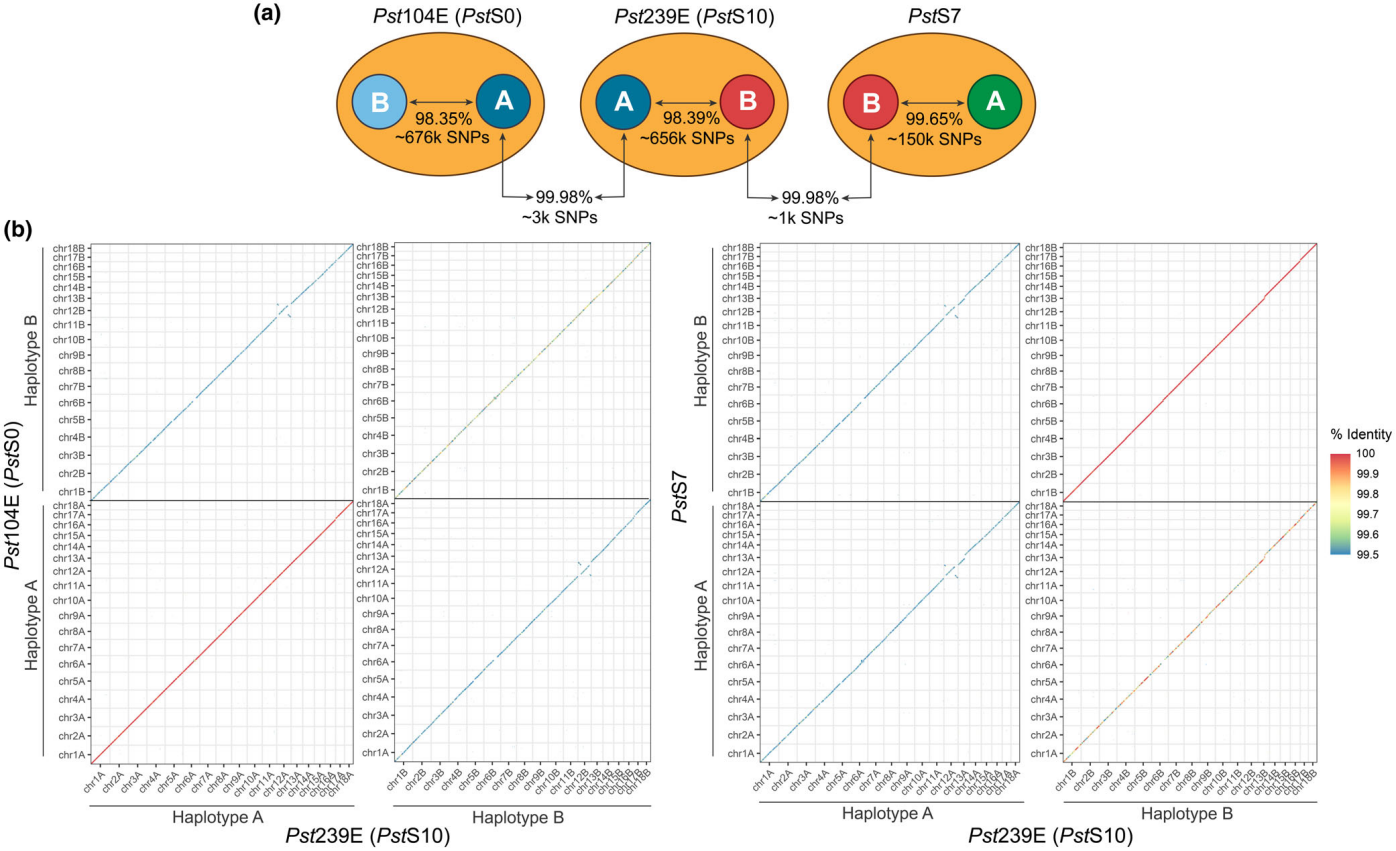
(a) Total single nucleotide polymorphism (SNP) counts and average identity from pairwise genome alignments among the nuclear haplotypes (denoted A and B) of clonal group PstS10 and two hypothetical donor nuclei from clonal groups PstS0 and PstS7, respectively. (b) Dotplots of all pairwise genome alignments among the nuclear haplotypes. Only alignment blocks with > 99.5% identity and longer than 10 kbp are shown.

The SSR haplotype results were confirmed by pairwise genome alignments and SNP counts derived from the nuclear‐phased haplotypes of the three isolates representing PstS0, PstS7 and the first detected MLG of PstS10, respectively (Fig. 4). Nucleus A in the hybrid (PstS10) displayed a 99.98% similarity with the corresponding nucleus A in PstS0 based on whole‐genome alignments with only *c*. 3 k SNPs differences when compared with intra‐isolate nuclear similarities of < 98.35% with over 650 k SNPs in PstS0. Similarly, the nucleus B of the hybrid displayed 99.98% similarity with the corresponding nucleus in PstS7 with *c*. 1 k SNPs when compared with over 150 k SNPs between the two nuclei within PstS7.

An overlay of allele sizes of the PstS7 haplotypes on PstS13 and PstS14 suggested that the PstS7 nucleus B was likely involved in hybridization that resulted in these clonal groups. Furthermore, the genome‐anchored SSR haplotype data suggested that PstS1_2 was the donor of the second nucleus for PstS14 (Fig. S4; Table S3).

### Long‐distance dispersal events

**Fig. 5:**
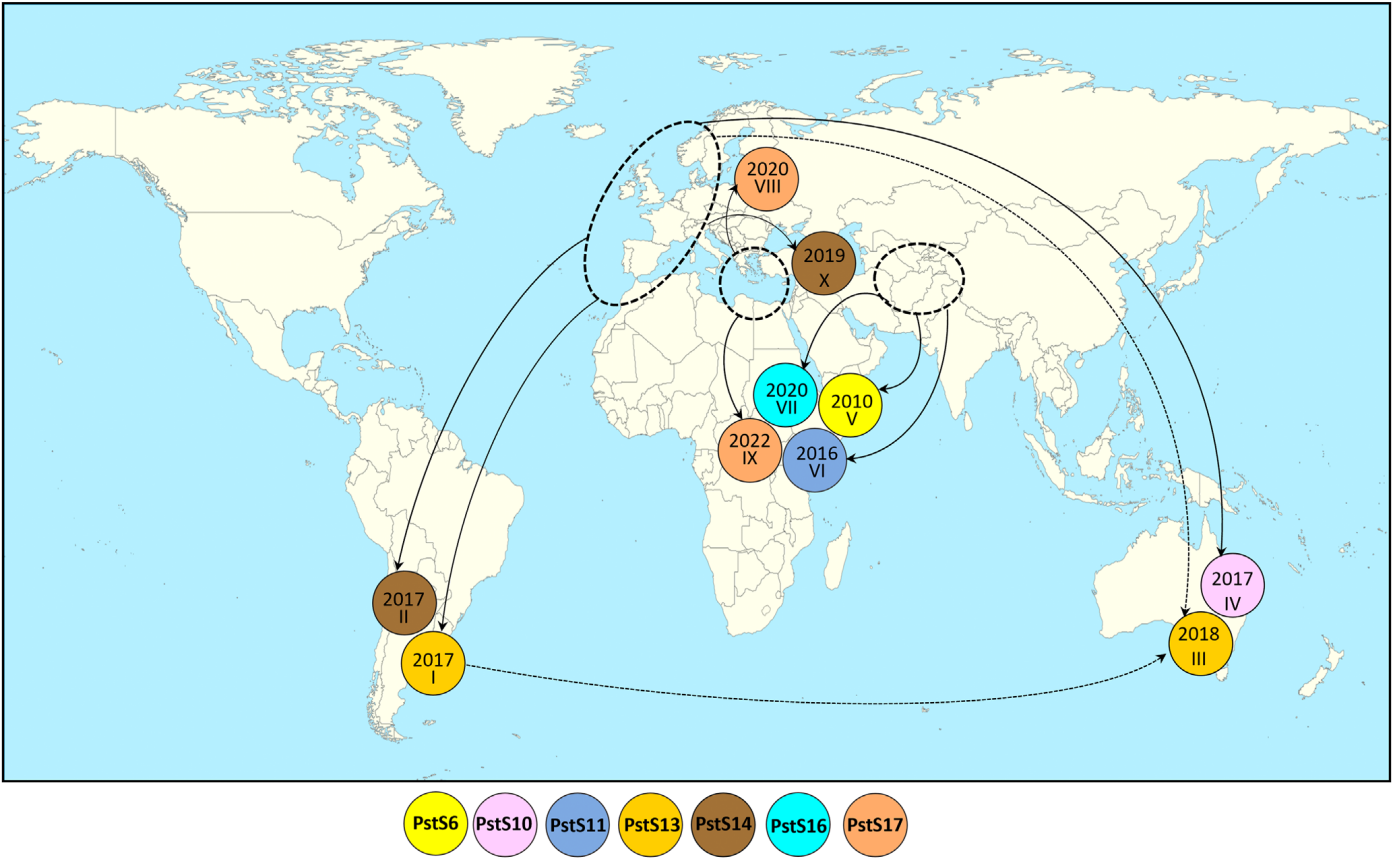
Documented incursions of *Puccinia striiformis* 2009–2023 based on sequential resampling of MLGs/pathogen races on different continents. Dotted circles represent the putative source areas, where considered *Pst* clonal groups defined by SSR markers were first detected, typically 2–3 yr ahead of first detection in recipient areas. Putative transmission routes are indicated by black arrows, and colored circles represent first year of detection on recipient continent and corresponding dispersal events. Dotted arrows: Clonal group PstS13 was present at two continents at time of incursion into Australia. The positions of circles and arrows on the map are indicative.

Ten cases of *Pst* incursions at intercontinental scales (I–X) were documented by shared MLGs and shared virulence phenotype in case alive isolates were available for testing (Table 1; Fig. 5). PstS13 that was first detected in Europe in 2015 (MLG #21) spread into South America in 2017 and Australia in 2018 (Table 1; dispersal events I and III). Recovered samples from South America (2017) shared virulence phenotype with a representative sample of the MLG (#21) in Europe. In 2017, PstS14 was detected for the first time outside of Europe/NW Africa (South America, dispersal events II), and PstS10 was first detected in Australia (dispersal event IV).

PstS6 (MLG #8), PstS11 (MLG #78) and PstS16 (MLG #205) that were first detected in Central/South Asia in 2009, 2012 and 2017, respectively, were observed for the first time in East Africa in 2010, 2015 and 2020, respectively (Events V, VI, VII; Table 1). PstS17 (MLG #170), first detected in the Middle East in 2018, was detected in northern Europe in 2020 (Event VIII; Table 1) and East Africa in 2022 (event IX). Identical virulence phenotype based on pairs of recovered samples representing source and recipient areas for dispersal events V‐IX confirmed a shared, common origin in each of these cases. PstS14 (MLG #138) was first detected in the Middle East in 2019 (Event X; Table 1).

## Discussion

This study represents one of the most extensive spatiotemporal surveys of a major crop pathogen to date (cf. Savary *et al*. (2019)). Sampling across 41 countries on six continents involved more than 400 collaborators (Table S6). Regular sampling spanning across 15 yr in Africa, Asia, Europe and South America became connected to long‐term surveillance activities in Australia through key reference samples from the first yellow rust appearance on the continent and until recent times (1978–2021). North American samples were fewer and more sporadic, for example, from the historic Stubbs collection (Thach *et al*., 2015), epidemiological studies on pathogen adaptation to warmer temperatures and host resistance (Milus *et al*., 2006, 2009, 2015b), and a recent Canadian study (Holden *et al*., 2025). Altogether, this allowed us to explore fundamental hypotheses about the evolutionary origin of newly detected clonal groups in *Pst*, as well as LDD dispersal capacity considering current insights about pathogen diversity and dynamics at regional and global scales.

### Somatic hybridization by nuclear swap between hypothesized parental isolates

Our study provides multiple, independent sets of data, which are consistent with somatic hybridization via nuclear reassortment as driver of evolution of several clonal groups (PstS10, PstS13, PstS14 and PstS18). Nuclear haplotype A of a prevalent European race (a.k.a. Solstice‐Oakley) in PstS0 (MLG #280) provided an almost 100% match with a nuclear haplotype of the first detected and most prevalent MLG (#244) of PstS10, whereas the second haplotype (B) provided a similar match with a nuclear haplotype represented by a race (a.k.a. Warrior) of the most prevalent MLG in PstS7 (#321) in the study. Independent genome anchored SSR haplotype data based on regular sampling during the 15‐yr study were consistent with this conclusion by providing a 100% match between corresponding SSR haplotypes of hypothesized parental isolates and a resulting hybrid. Interestingly, our genome anchored SSR haplotype data also suggested that PstS13 and PstS14 emerged by nuclear reassortment between co‐existing parental pairs of isolates, initially suggested by compatible allele sizes (Tables S3, S5). In the case of PstS14, the SSR haplotype data suggested that PstS7 (nucleus B) and PstS1_2 (nucleus A) formed the PstS14 hybrid, supported by a blast of SSR primer sequences to the chromosome‐scale assembly of a PstS1_2 representative isolate (Table S3, Fig. S4; Schwessinger *et al*., 2022). This was further supported by independent epidemiological observations in Northwest Africa, the only area and time, where PstS1_2 and PstS7 co‐existed before first detection of PstS14 (illustrated by Fig. 2). Interestingly, PstS14 made up 100% of samples in surveys in three consecutive years in NW Africa after first detection, that is 2016 (six samples), 2017 (38 samples) and 2018 (54 samples), where only observed sporadically elsewhere.

An overlay of the PstS7 SSR haplotypes on the PstS13 genotype showed that PstS7 nuclear haplotype B was shared, whereas haplotype A was excluded due to multiple allele‐size mismatches (data not shown). The derived SSR haplotypes of PstS13 nucleus A matched perfectly to the most prevalent SSR genotype in PstS4 (MLG #38, 72% of samples), resulting in a 100% allelic match based on the 16 primer pairs that produced a positive genome blast result (Table S7). The existence of exclusive and shared SSR alleles for isolates of PstS13 and PstS4 gave further support for this hypothesis (e.g. RJN11, allele size 172). Before first detection of PstS13, PstS7 was frequently detected on both wheat and triticale in northern Europe (2011–2014), whereas PstS4 was mainly detected on Triticale and rarely bread wheat (2008–2014; https://agro.au.dk/forskning/internationale‐platforme/wheatrust/yellow‐rust‐tools‐maps‐and‐charts/genetic‐groups‐frequency‐map). In summary, this suggests that a hybridization resulting in PstS13 (MLG #21) most likely took place on Triticale, however, resulting in a significant impact on *Pst* epidemiology on a wider panel of crops, including durum wheat and spring wheat in particular (Anibal Carmona *et al*., 2019; Ding *et al*., 2021).

**Fig. 6:**
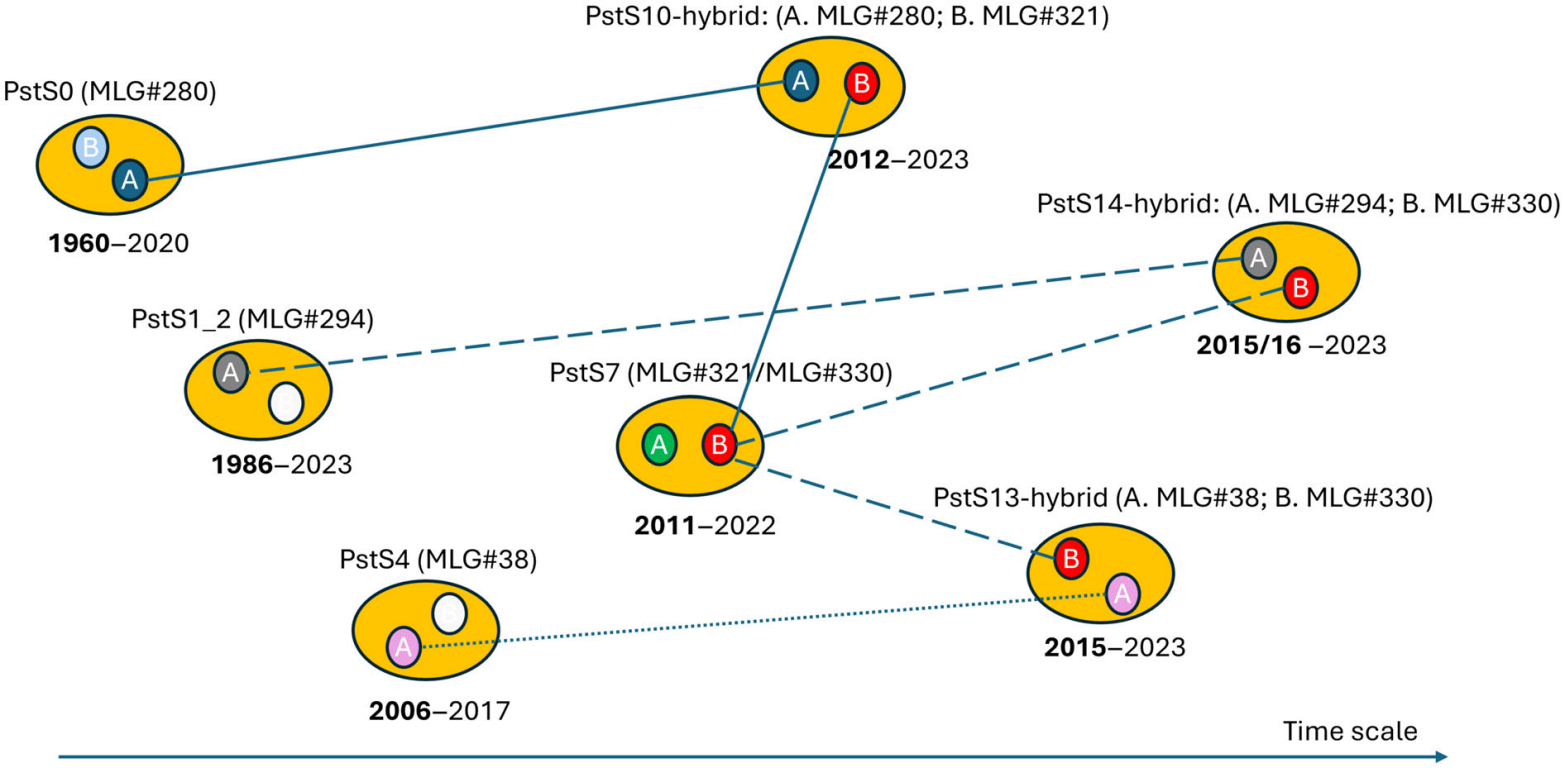
Schematic representation of three hypothesized *Puccinia striiformis* hybridization events supported by fully phased genomic data (solid lines), chromosome‐scale genome anchored SSR haplotype data (long‐dash lines), and derived SSR haplotype data (dotted line). The two nuclear haplotypes within individuals are denoted A and B, respectively. Clonal group PstS10 was first detected in northern Europe (2012) in an area where the respective MLGs of PstS0 (#280) and PstS7 (#321) were prevalent on shared hosts of bread wheat. Clonal group PstS13 was first detected in 2015, where a single MLG (#21) was detected at multiple locations in Europe, often on Triticale and rarely on bread wheat. The involved MLG of clonal group PstS4 (#38) was mainly detected on Triticale, while the MLG of clonal group PstS7 (#330) was detected widely on triticale as well as bread wheat. Clonal group PstS14 was first detected in 2015, the involved MLGs of clonal groups PstS7 (#330) and PstS1_2 (#294) being prevalent in southern Europe/NW Africa at that time. First and last sampling year of individual genetic groups in the dataset are indicated (years before 2009 represented by historic reference samples).

A schematic representation of the hybridization events resulting in PstS10, PstS13 and PstS14 is summarized in Fig. 6.

These conclusions are consistent with the preliminary reporting of likely asexual hybridization in *Pst* in North America, involving a PstS1_2 isolate and a second unknown parent that may have resulted in PstS18 (Holden *et al*., 2025). PstS18 was defined recently by Thach *et al*. (2025) with the earliest PstS18 samples originating from five different locations in Arkansas (USA) in 2013 (Milus *et al*., 2015b), followed by two samples provided by CIMMYT in 2023. Interestingly, our SSR haplotype data confirm PstS1 (denoted PstS1_2 in the current study) as donor of one of the nuclei in PstS18 (Table S7). This is further supported by the presence of a unique diagnostic SCAR marker for PstS1 (Walter *et al*., 2016). The SSR data from a different US sample revealed a complete match of unique allele sizes that can identify the donor of the second parental nucleus of PstS18. This isolate was first detected in 2007 according to Milus *et al*. (2015b). All together, these observations suggest that PstS18 indeed is a somatic hybrid emerging from two parental isolates that were already present in the United States in 2013, where the first documented case of PstS18 was observed.

Although sexual reproduction cannot be categorically excluded, it appears highly unlikely in the present study. Completion of the sexual cycle in *P. striiformis* in nature has been rarely documented, for example based on extensive surveys of rust species on the sexual host, *Berberis* spp., and not outside of China (Berlin *et al*., 2013; Wang *et al*., 2016; Rodriguez‐Algaba *et al*., 2022). Second, phased SSR markers located on multiple chromosomes would be expected to reassort randomly following meiosis; the probability of recovering the observed multilocus configuration by chance is extremely low. Finally, in the case of PstS0 and PstS7, resulting in PstS10, the fully haplo‐phased genomic data exclude the possibility of haplotype reassortment, which is supported by the limited fertility of PstS0 isolates (Ali *et al*., 2010).

Our results represent previously undocumented aspects of yellow rust epidemiology suggesting that somatic hybridization may be a more frequent source of agriculturally relevant genetic diversity in wheat yellow rust than sexual recombination. Unlike other cereal rust fungi, somatic hybridization in *Pst* involving nuclear reassortment has only been reported under experimental conditions. Already in the 1960s, mixtures of urediniospore isolates of different races suggested that somatic hybridization by fusion of hyphae and subsequent nuclear reassortment was the likely source of new race variants (Little & Manners, 1967, 1969). More recently, cross‐infection studies resulting in 68 experimental isolates supported the occurrence of nuclear reassortment in one case, whereas most results were explained by assuming additional chromosome reassortment and crossovers (Lei *et al*., 2017). Earlier, Park & Wellings (2012) concluded that somatic hybridization was likely in many rust species and *formae speciales*, the process being observed in nature within and between rust species on *Linum*, *Populus*, *Senecio*, several grass species and leaf rust on wheat. However, the exact mechanisms are generally not well understood.

### Long‐distance dispersal events

The potential impact of rare events of LDD of plant pathogens on plant health has been known for decades; for example, Brown & Hovmøller (2002), Smart & Fry (2001), Ristaino *et al*. (2021), Jones (2021). The focus has often been on high‐impact examples; for example, an overview for *Pst* on wheat by Jin *et al*. (2020), rather than cases with insignificant impact, which may be overlooked and thereby contribute to an underestimation of the true frequency of LDD in crop pathogens. In this study, hypothesized migration events at the intercontinental scale (LDD) were investigated by analyses of first detection of new clonal groups and the distinct MLGs within them in eight geographically separated populations.

In seven cases, these LDD events resulted in severe and widespread disease epidemics in recipient areas, for example PstS13 in South America (Anibal Carmona *et al*., 2019; Riella *et al*., 2024) and PstS10 and PstS13 in Australia (https://groundcover.grdc.com.au/weeds‐pests‐diseases/diseases/stripe‐rust‐incursions‐create‐huge‐challenges; Ding *et al*., 2021), whereas three cases had only minor epidemiological impact (i.e. rare detection of corresponding clonal groups in following years). Our conclusions concerning the hypotheses of LDD vs an alternative hypothesis of hybridization of ‘local’ isolates were supported by lack of detection of matching parental isolates in recipient areas, for example PstS7 in Australia and PstS1_2 and PstS4 in South America, despite extensive and widespread surveillance efforts (www.wheatrust.org; Anibal Carmona *et al*., 2019; Ding *et al*., 2021; Riella *et al*., 2024).

The conclusion of very recent dispersal events was supported by identical virulence phenotypes in source and recipient areas based on assays comprising wheat experimental lines representing more than 25 different host resistance specificities (Thach *et al*., 2025). Because mutation from avirulence to virulence in plant pathogenic fungi, including *Pst*, may be higher than average mutation rates (Hovmøller & Justesen, 2007), and the impact is likely accelerated by host‐induced selection (Wellings & McIntosh, 1990; Bayles *et al*., 2000; de Vallavieille‐Pope *et al*., 2012), the existence of an exact match in virulence phenotype indicates connectivity between such populations in a short‐term perspective.

Considering common source and recipient areas of the 10 reported LDD events, we developed exploratory analyses to check the plausibility of aerial spore dispersal by wind for seven putative transmission routes to complement our observational data, for example for providing a framework for in‐depth testing of hypotheses about specific dispersal events, pinpointing data requirements in terms of additional surveillance efforts in critical geographical areas (direct vs stepwise transmission), better estimates of spore survival times in the atmosphere, and more comprehensive simulations taking advantage of the rapidly increasing amount of available meteorological data and computing power. The analyses were based on dispersal distance, a combination of atmospheric trajectory simulations, high‐resolution global meteorological data, data on overlapping wheat growing seasons, for example Bradshaw *et al*. (2022), and testing spore survival times of 5 and 10 d in the atmosphere, respectively, based on indicative evidence from previous studies on related pathogens, for example Maddison & Manners (1972), Rapilly (1979), Aylor (1986).

Our results indicated that both human‐mediated and windborne dispersal may play an important role in intercontinental dispersal of wheat yellow rust (Table 2). Airborne dispersal appears especially plausible between the Middle East and East Africa as well as Europe. This aligns with observed genetic similarities between *Pst* populations in the Middle East and East Africa that also indicate frequent gene flow between these regions and help explain several of the reported incursion events. The recent detections of PstS6, PstS11, PstS16 and PstS17 in East Africa after previous detections in the Middle East and Central/South Asia provide supportive evidence for the previously proposed Rift Valley Incursion pathway for rust fungi (Meyer *et al*., 2017a). Direct dispersal from Central/South Asia to East Africa seems highly unlikely, but the possibility of stepwise airborne dispersal via the Middle East cannot be ruled out. Stepwise transmission is less supported by current data because PstS6 and PstS16 were not detected in the Middle East, and PstS11 appeared in the region only 3 yr after first detection in East Africa. However, due to a relatively low sampling intensity in critical areas on the Arabian Peninsula and the Horn of Africa and simulations indicating a plausible stepwise transmission, we cannot exclude this possibility.

**Table 2:** Plausibility of airborne dispersal of *Puccinia striiformis* along the dispersal routes identified from 15 yr of surveys across 41 countries in six continents. Plausiblity of windborne dispersal was assessed by combining data from: (A) previous studies of related pathogens in similar geographies, (B) analyses of long‐term monthly means on meteorological data on average wind direction and speed at times of overlapping wheat growing seasons in source and recipient areas; (C) trajectory simulations for tentative source locations and times and considering different maximum spore survival times. Color shading is used to distinguish heading and hypothesis from results.

Dispersal route	Route 1	Route 2	Route 3	Route 4	Route 5	Route 6	Route 7
Source Region	Europe	South America	Europe	Central/South Asia	Middle East	Middle East	Europe/North Africa
Target Region	South America	Australia	Australia	East Africa	Europe	East Africa	Middle East
Dispersal distance	d *c.* 9500 km	d *c.* 12 500 km	d *c.* 13 500 km	d *c.* 4000 km	d *c.* 3000 km	d *c.* 2800 km	d *c.* 3200 km
Observations indicate LDD: Migrants reported in this study^1^ ‐first detection year in source and target region	PstS13 (2015, 2017), PstS14 (2015, 2017)	PstS13 (2017, 2018)	PstS13 (2015, 2018), PstS10 (2012, 2017)	PstS6 (2009, 2010), PstS11 (2012, 2016), PstS16 (2017, 2020),	PstS17 (2018, 2020)	PstS17 (2018, 2022)	PstS14 (2016, 2019)
(A) Indicative evidence of airborne dispersal from previous studies on related pathogens^2^	Direct transmission not plausible [4] Transport of biological particulates over the Atlantic Ocean, for example [1], [2], [3]	Direct transmission not plausible [4]	Human‐mediated dispersal hypothesized [3, 5] No previous dispersal simulations	Direct transmission very unlikely but possible [6, 7] Stepwise transport from C/SA via ME to EA plausible [6, 7]	Direct transmission plausible [6, 7]	Direct transmission plausible[6, 7, 8]	Direct transmission plausible[4, 9]
(B) Long‐term trends in wind direction & speed	Direct transmission not plausible Stepwise transmission might be plausible	Direct transmission not plausible	Direct transmission not plausible	Direct transmission might be plausible Stepwise transmission plausible	Direct transmission plausible	Direct transmission plausible	Direct transmission plausible
(C) Atmospheric trajectory simulation outputs^3^	No backwards trajectories from SA reach EU even for 10‐d lifetime Forward trajectories indicate stepwise transmission may occur albeit being extremely rare	Individual backwards trajectories from AUS reach SA with 10‐d lifetime, but none for 5 d	No backwards trajectories from AUS reach EU even with 10‐d lifetime	No backwards trajectories for Ethiopia reach CA/SA at 10‐d lifetime Individual backwards trajectories from Yemen reach southern India with 10‐d lifetime, none for 5‐d lifetime Forward trajectories support stepwise transmission	Backwards trajectories from EU reach ME	Backwards trajectories from EU reach ME	Backwards trajectories from ME reach EU/NA
Hypotheses	Direct windborne dispersal not plausible. Stepwise dispersal not excluded	Direct or stepwise windborne dispersal not plausible	Direct or stepwise windborne dispersal not plausible	Direct windborne dispersal may be plausible. Stepwise windborne dispersal plausible when considering previous modelling studies but not supported by observations	Direct windborne dispersal plausible	Direct windborne dispersal plausible	Direct windborne dispersal plausible

^1^ Denoted by clonal group hosting considered migrant MLG (year of first detection at source, year first detection target).

^2^ References: [1] Brodsky *et al*. (2023), [2] Isard *et al*. (2011), [3] Hovmøller *et al*. (2002), [4] Prank *et al*. (2019), [5] Miedaner & Garbelotto (2024), [6] Meyer *et al*. (2017a), [7] Meyer *et al*. (2017b), [8] Bradshaw *et al*. (2022), [9] Radici *et al*. (2022).

^3^ Sets of backwards & forward trajectory simulations were conducted using HYSPLIT with GDAS and ERA5 data for developing hypotheses about the plausibility of airborne transmission.

Direct airborne dispersal from Europe to South America or from Central/South Asia to East Africa is highly unlikely, but we hypothesize that indirect stepwise windborne transmission might have occurred, noting that a more comprehensive modelling study is required to test this. For the two dispersal events from Europe to Australia and South America to Australia, wind dispersal was not plausible, strongly indicating the role of human‐mediated transport and emphasizing the importance of quantifying risks due to unintended human‐mediated transmission via trade and travel.

Altogether, our study has documented a high degree of connectivity world‐wide between populations of one of the most epidemic crop pathogens, yellow rust infecting cereals and grasses. Incursions from the Himalayas and elsewhere into Europe and the Mediterranean basin 10–20 yr ago (Hubbard *et al*., 2015; Hovmøller *et al*., 2016; Walter *et al*., 2016) hybridized with isolates of pre‐existing genetic groups, for example PstS0, PstS1_2, and PstS4, the new hybrids to a large extent replaced existing population(s), spreading onwards to new continents (Hovmøller *et al*., 2023), where they rapidly became dominant (Anibal Carmona *et al*., 2019; Ding *et al*., 2021; Riella *et al*., 2024). The connectivity between populations of airborne pathogens is probably particularly evident in global cropping systems like wheat, which is grown on six continents.

Our data suggest that the risks and pace of pathogen evolution driven by mutation, somatic hybridization and selection may correlate positively with the size and areas affected by ongoing epidemics. In turn, this may influence the probabilities of further hybridization and risks of unintentional pathogen spread at local, regional and global levels. Thus, there is an imminent and urgent need to sustain pathogen surveillance to better anticipate and prevent future epidemics and to facilitate development of resistant wheat cultivars. In parallel, greater awareness is needed around the risks of unintentional crop pathogen spread via human activity to enhance food security.

## Competing interests

None declared.

## Author contributions

DH and MSH conceived and designed the surveillance program. TT carried out the experimental SSR genotyping work under the supervision of AFJ, including data alignment and quality check and population genetic analyses. VR assisted in genotyping of samples from South America. JRA performed virulence phenotyping of putative migrant isolates and assisted in SSR genotyping and data analyses. JGH developed the web‐based data management and display system for world‐wide prevalence of *Pst* genetic groups. KN supplied raw SSR data for Middle East samples 2018–2020. RP was curator of reference samples representing the Australian *Pst* population 1979–2021, undertook race analysis and was responsible for associated funding. RT and MM performed the sequencing and genomic data analyses of PstS10 hybrid and parental isolates. BS and JR contributed to genomic data analysis, supervised and secured funding for the genomic work and provided extensive feedback on the manuscript. PS supervised the PhD work of VR in South America. MME conducted trajectory simulations of the plausibility of wind dispersal of spores based on meteorological datasets. MSH was curator of samples hosted by the GRRC, supervised project activities anchored at GRRC, wrote drafts of the manuscript. All authors revised and accepted the final manuscript. MSH and TT contributed equally to this work and shared first authorship.

## Disclaimer

The New Phytologist Foundation remains neutral with regard to jurisdictional claims in maps and in any institutional affiliations.

## Supporting information


**Fig. S1** Geographical sampling areas and number of samples per country across six continents.
**Fig. S2** Genotypic resolution based on 18 polymorphic SSR markers with assigned chromosomal location.
**Fig. S3** Approach for exploring hypotheses of hybridization events in *Pst*.
**Fig. S4** SSR marker placement in *Puccinia striiformis* clonal group PstS1 (race 134E).

**Table S1** World‐wide collection of 3240 *Puccinia striiformis* samples from 41 countries and six continents grouped according to geographical sampling origin.
**Table S2** Genome assembly quality statistics.
**Table S3** Calculated amplicon length and chromosome location of SSR primers based on haplo‐phased genome information of representative PstS0, PstS7, PstS10 and PstS1 isolates.
**Table S4** SSR diversity in *Puccinia striiformis* clonal groups defined according to Thach *et al*. (2025).
**Table S5** Number of allele‐size mismatches at 19 SSR loci for parental isolates of clonal groups hypothesized to result in PstS10, PstS13 and PstS14, respectively.
**Table S6** Overview of 419 people contributing to world‐sampling.
**Table S7** Calculated and scored allele sizes of haplotypes of putative parental and resulting hybrids represented by PstS13, PstS14 and PstS18.
Please note: Wiley is not responsible for the content or functionality of any Supporting Information supplied by the authors. Any queries (other than missing material) should be directed to the *New Phytologist* Central Office.

## Data Availability

All raw sequencing data generated in this study have been submitted to NCBI BioProject database under accession no.: PRJNA1256629. Project accessions for Pst239E (PstS10) are as follows: PRJNA1260730: *Puccinia striiformis* f.sp. *tritici* haplotype 1 genome sequencing (haplotype A), PRJNA1260729: *Puccinia striiformis* f.sp. *tritici* haplotype 2 genome sequencing (haplotype B); PstS7: PRJNA1260732: *Puccinia striiformis* f.sp. *tritici* haplotype 1 genome sequencing (haplotype A), PRJNA1260731: *Puccinia striiformis* f.sp. *tritici* haplotype 2 genome sequencing (haplotype B). Genome assemblies are also available at doi: 10.5281/zenodo.18396413. The SSR genotypic data are available at doi: 10.5281/zenodo.15481965.
