# Supplementary material for: Long‐term surveillance reveals hybridization by nuclear reassortment and intercontinental spread as major evolutionary drivers in wheat yellow rust
Source: New Phytol. 2026 Jun 14;251(4):2091–106. doi: 10.1111/nph.71300 (PMC13373815; doi:10.1111/nph.71300)

New Phytologist

New Phytologist **Supporting Information - Figures**

Article title: **Long-term surveillance reveals hybridization by nuclear reassortment and intercontinental spread as major evolutionary drivers in wheat yellow rust**

Authors: Mogens Støvring Hovmøller, Tine Thach, Julian Rodriguez-Algaba, Jens Grønbech Hansen, Marcel Meyer, David P. Hodson, Kumarse Nazari, Robert F. Park, Rita Tam, Mareike Möller, Benjamin Schwessinger, John Rathjen, Paula Silva, Venancio Riella and Annemarie Fejer Justesen

Article acceptance date: 09 May 2026

The following Supporting Information is available for this article:

Fig. S1 Geographical sampling areas and number of samples per country across six continents.

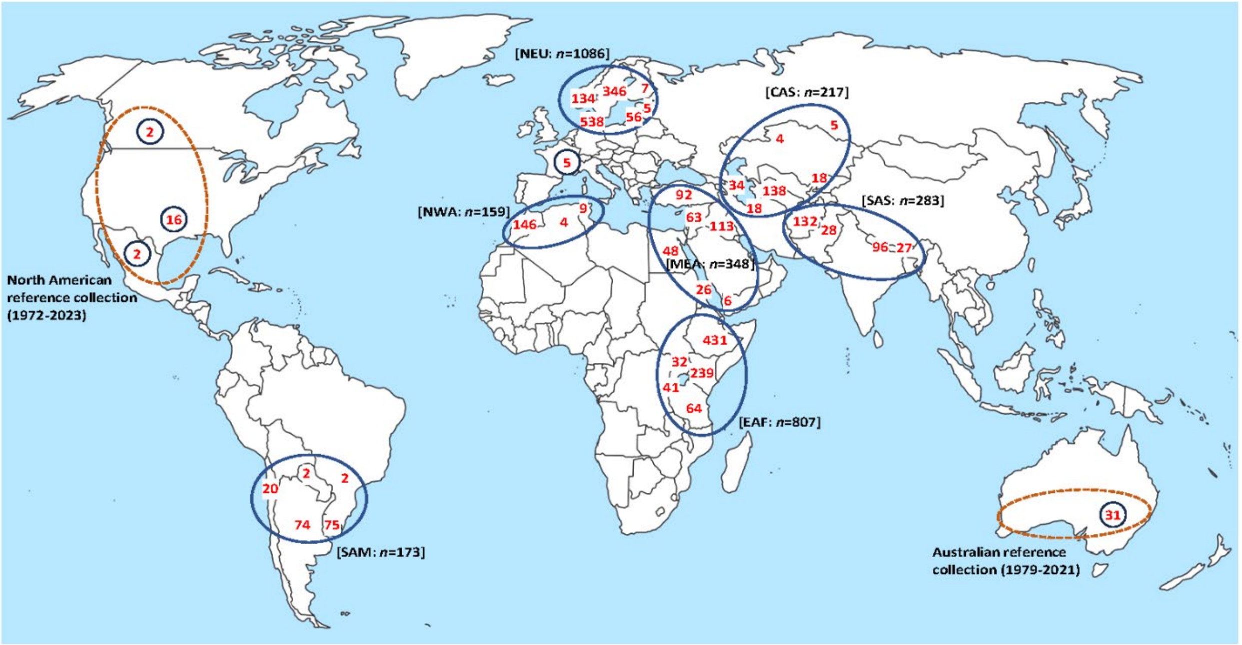

Fig. S2 Genotypic resolution based on 18 polymorphic SSR markers with assigned chromosomal location.

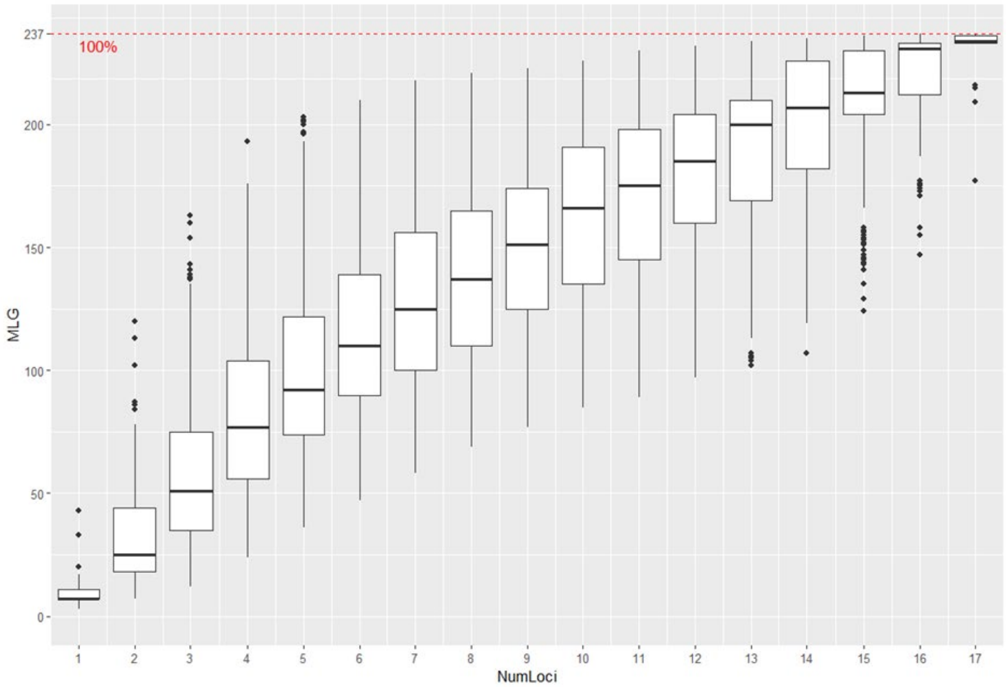

Figure S3 Approach for exploring hypotheses of hybridization events in Pst based on fully phased genome assemblies, chromosome-scale genome anchored SSR haplotypes, and inferred SSR haplotypes derived from such data. In the present study we focused on three novel, clonal groups that were first detected in the dataset in 2012 (PstS10), 2015 (PstS13) and 2015 (PstS14), respectively.

1. Comparisons of allele sizes of new MLGs representing novel groups with pre-existing clonal groups in the dataset

⇒ MLGs with allele sizes (including null alleles) that did not share allele sizes with a novel MLG were excluded as potential parents in hypothesized hybridization by nuclear reassortment (Table S5).

↓

2. Determination of nuclear-specific SSR haplotypes and genomic validation of PstS10 hybrid

a. SSR haplotypes of hypothesized parental isolates (PstS0 and PstS7) contributing to PstS10 (according to step 1) were determined by blasting SSR primer sequences to chromosome-scale haplophased genomic data of PstS0 and PstS7 resulting in fully phased SSR haplotypes (Fig. 3)
b. Validation of PstS10 SSR haplotypes by blasting SSR primer sequences to chromosome-scale haplophased genome of PstS10 (Fig. 3).
c. Validation of nuclear reassortment by SNP counts and average identity based on pairwise genome alignments (Fig. 4).

↓

3. Determination of nuclear-specific SSR haplotypes of parental pair resulting in the PstS14 hybrid

a. Following step 1-2, PstS1_2 and PstS7 were tested as potential parents of the first detected MLG of PstS14 (cf. Table S3). Haplotype A of PstS7 was excluded due to mismatching allele sizes. The genotype of PstS14 and haplotype B of PstS7 revealed the 2nd SSR haplotype of PstS14 (likely originating from PstS1_2).
b. Validation of SSR haplotypes of PstS1_2 by blasting of SSR primer sequences to chromosome-scale haplophased genome of PstS1_2 resulting in fully phased SSR haplotypes confirmed PstS1_2 as one of the parents (Fig. S4).
c. Based on the global SSR genotype data set both parental haplotypes that resulted in 100% match of the first observed MLG of PstS14 were confirmed to co-exist in NW Africa 2013-2014.

↓

4. SSR haplotype inference of parental pair resulting in the PstS13 hybrid

a. Following step 1-2, PstS4 and PstS7 were tested as potential parents of the first detected MLG of PstS13 (cf. Table S3). Haplotype A of PstS7 was excluded due to mismatching allele sizes. The genotype of PstS13 and haplotype B of PstS7 revealed the 2nd haplotype of PstS13 (likely originating from PstS4).
b. Validation: Allele sizes of hypothesized parental haplotypes, which co-existed on Triticale in northern Europe 2011-2013, resulted in 100% match of the first observed MLG of PstS13 (2015).

Figure S4 SSR marker placement in P. striiformis clonal group PstS1 (race 134E)

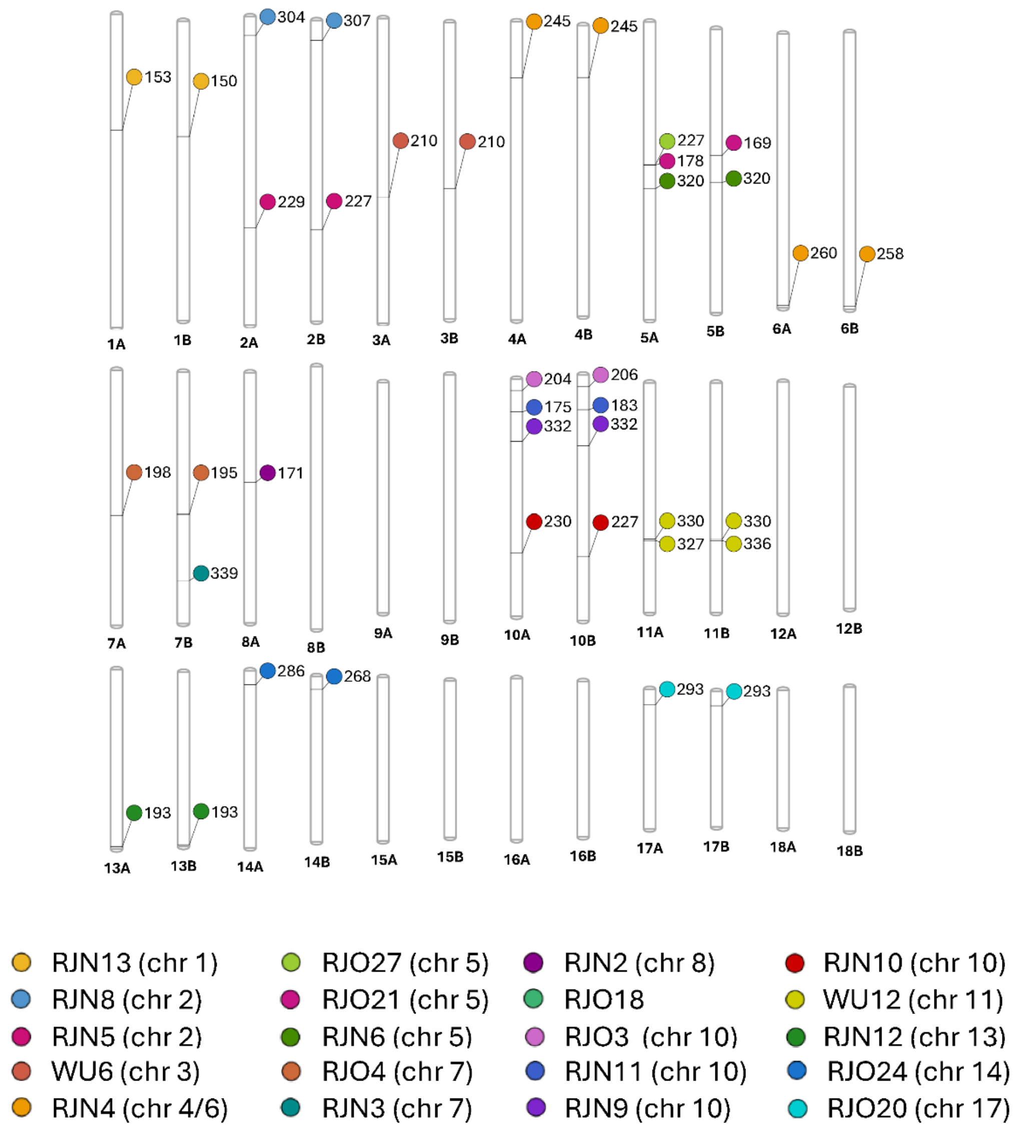

Supplement: Supplementary file 1 — Fig. S1 Geographical sampling areas and number of samples per country across six continents. Fig. S2 Genotypic resolution based on 18 polymorphic SSR markers with assigned chromosomal location. Fig. S3 Approach for exploring hypotheses of hybridization events in Pst. Fig. S4 SSR marker placement in Puccinia striiformis clonal group PstS1 (race 134E). [file NPH-251-2091-s001.pdf]
